# Can artificial intelligence uncover the bioactive peptides’ benefits for human health and knowledge? A narrative review

**DOI:** 10.3389/fnut.2025.1698147

**Published:** 2025-12-15

**Authors:** Rolan Al Shareef, Eihab Fathelrahman, Raeda Osman, Tamrat Gebiso, Carine Platat

**Affiliations:** 1 Department of Nutrition and Health, College of Medicine and Health Sciences, United Arab Emirates University (UAEU), Al Ain, United Arab Emirates; 2 Department of Integrative Agriculture (INAG), College of Agriculture and Veterinary Medicine (CAVM), United Arab Emirates University (UAEU), Al Ain, United Arab Emirates

**Keywords:** bioactive compound, bioactive peptides, food sources, artificial intelligence, machine learning, deep learning, health and knowledge benefits, databases

## Abstract

The intersection of Artificial Intelligence (AI) and food science has opened new frontiers in understanding the “dark matter” of food, the vast array of unidentified bioactive compounds that influence human health. This narrative review examines how AI, particularly machine learning and deep learning, is revolutionizing the discovery, characterization, and application of bioactive peptides and amino acids derived from food sources, both plant- and animal-based. These compounds exhibit diverse health benefits, including antioxidant, anti-inflammatory, antihypertensive, and antimicrobial properties, yet their complexity and the limitations of traditional methods have hindered comprehensive study. AI-driven approaches, such as predictive modeling, molecular dynamics simulations, and natural language processing, are accelerating the identification of bioactive peptides, optimizing extraction processes, and enabling personalized nutrition strategies. The integration of AI with omics technologies (e.g., nutrigenomics, proteomics) further enhances our understanding of how these peptides modulate physiological pathways. However, this is not without challenges and limitations, such as data quality, model interpretability, and persistent gaps in interdisciplinary collaboration. Additionally, the review highlights the lack of standardized databases and concerns about the use of AI, including the need for ethical approvals and protocols aligned with privacy laws, particularly in the context of personalized nutrition guidance. This review synthesizes current advancements, identifies research gaps, and underscores the transformative potential of AI in functional food development and precision nutrition. By addressing these challenges, AI can unlock the full therapeutic potential of food-derived bioactive compounds, providing innovative solutions to global health challenges such as non-communicable diseases. The findings advocate for robust interdisciplinary efforts to bridge computational and nutritional sciences, paving the way for scalable, evidence-based applications in health and wellness.

## 1 Introduction

In metabolomics, the term “dark matter” denotes the large fraction of spectral features that cannot be confidently assigned to known molecular entities (1). Current estimates suggest that over 90% of the detectable chemical space in foods remains structurally uncharacterized, forming the dark matter of the food metabolome and, more broadly, of the dietary exposome (2, 3). This unresolved domain encompasses both genuine but unidentified biomolecules and redundant or artefactual signals that inflate the apparent feature count. The lack of a robust operational framework to distinguish between these categories constrains efforts to uncover the molecular basis of diet–health interactions (1, 2). Resolving this chemical dark matter is thus essential to bridge the gap between compositional profiling and functional interpretation, enabling a deeper understanding of how food components influence human physiology (1, 3). Despite substantial advances in analytical chemistry, the complexity and scale of untargeted metabolomic data continue to exceed the capacity of conventional annotation workflows (2). This persistent gap underscores the need for new computational paradigms capable of discerning meaningful patterns within high-dimensional spectral datasets (4).

Artificial intelligence (AI) offers a powerful and transformative framework for identifying bioactive compounds in complex food matrices (5). By integrating machine learning (ML), a subset of AI focused on pattern recognition and prediction and molecular networking with spectral interpretation, AI systems can systematically prioritize unknown features, predict structural motifs, and infer potential bioactivities (3, 6, 7). Such computational approaches hold promise for elucidating bioactive fractions, particularly food-derived peptides and amino acid derivatives that underpin the molecular determinants of food bioactivity and human health (3, 7). These bioactive compounds represent some of the most functionally significant yet analytically complex components of the food metabolome, exhibiting diverse physiological effects including antioxidant, anti-inflammatory, antimicrobial, and immunomodulatory activities (8, 9).

Bioactive peptides are specific amino acid sequences (typically 2–20 residues) encrypted within food proteins and released through enzymatic hydrolysis during digestion or processing (7, 10). Their biological activity depends on sequence composition, length, conformation, and post-translational modifications, which collectively determine target affinity, stability, and bioavailability. Representative examples include antihypertensive peptides that inhibit angiotensin-converting enzyme (ACE), opioid-like peptides that modulate gut–brain signaling, and antioxidant peptides that scavenge reactive oxygen species (10, 11). The functional outcomes of these peptides are sequence- and dose-dependent and require validation through physiologically relevant *in vitro* and *in vivo* models. In contrast, free amino acids such as glutamine, arginine, and branched-chain amino acids (BCAAs) act through distinct metabolic and signaling pathways, influencing nitrogen balance, immune modulation, and muscle repair (3, 7, 12). Despite their demonstrated benefits, the identification and characterization of these bioactive compounds remain constrained by the complexity of food matrices and the vastness of their underlying chemical space (11, 13).

AI encompassing ML and deep learning (DL), a class of neural-network-based methods capable of automatically learning hierarchical representations from data, has begun to address these limitations through task-specific modeling frameworks that enhance the discovery and validation of food bioactives (5, 14). In modern peptidomics, large-scale datasets generated from liquid chromatography–mass spectrometry (LC–MS) analyses, peptide sequences, and physicochemical descriptors can be mined using supervised and unsupervised ML approaches to prioritize peptide sequences, predict digestive stability and intestinal transport, map proteins to peptides based on protease specificity, and estimate receptor-binding affinities (5, 12–14). DL architectures, including convolutional neural networks (CNNs) and recurrent neural networks (RNNs), have proven particularly effective in disambiguating LC–MS features, identifying functional motifs, and inferring peptide–receptor interactions from sequence and structural descriptors (3, 15).

Building on these foundations, emerging sequence-to-function models employ transformer architectures, advanced neural networks initially developed for natural language processing, to predict peptide bioactivity. These models are trained on peptide libraries annotated for activity endpoints, including antioxidant, ACE-inhibitory, and antimicrobial potency, enzymatic cleavage profiles, and intestinal transport metrics (16). Amino acid sequences are converted into tokenized embeddings capturing physicochemical, evolutionary, and contextual information through pretraining–fine-tuning pipelines; attention mechanisms and masked sequence prediction enable partial interpretability of model outputs (12, 13, 15, 16). Integrative quantitative structure–activity relationship (QSAR) models further enhance interpretability by linking sequence-derived descriptors to experimentally validated endpoints such as ACE inhibition or antioxidant capacity, thereby improving mechanistic insight and candidate prioritization (12, 13, 17). Complementary molecular dynamics (MD) simulations and docking analyses are often used to assess conformational stability, receptor interactions, and binding energetics, thereby supporting the experimental validation of top-ranked peptide candidates (3, 15, 16).

Model performance is quantitatively evaluated using metrics such as accuracy, precision, recall, F1-score, area under the receiver operating characteristic curve (AUROC), and area under the precision–recall curve (AUPRC) to ensure predictive reliability and reproducibility across laboratories (5, 14). Rigorous validation practices, such as cross-validation with sequence-homology filtering, external benchmarking, and cross-matrix testing, can help prevent information leakage and overfitting, particularly in heterogeneous datasets spanning multiple food matrices (12–14).

Recent applications demonstrate the translational potential of these AI-driven frameworks. For example, AI-guided screening has facilitated the identification of antihypertensive peptides from milk proteins and antioxidant peptides from soy and marine sources, where computational prioritization significantly narrowed the pool of candidates for synthesis and experimental verification (5). Such integrative methodologies enable the rational design and experimental prioritization of bioactive peptides, accelerating the translation of computational predictions into nutritionally and therapeutically relevant outcomes (3, 4, 6, 18–20). Collectively, AI-driven frameworks bridge compositional profiling with functional inference, enabling a more efficient, data-informed exploration of the food proteome.

However, persistent challenges constrain the broader adoption of AI in food peptide discovery. These include class imbalance in training datasets, cross-matrix extrapolation errors, scarcity of well-characterized negative samples, assay and database biases, limited mechanistic transparency in deep learning models, and restricted portability across species or food matrices (21). Moving forward, integrating multi-omic data and linking peptidomic profiles to metabolomic features, microbial transformations, and host physiological responses will be critical. This integration requires adherence to the FAIR principles (Findable, Accessible, Interoperable, and Reusable) that underpin reproducible research and facilitate meaningful biological interpretation. When incorporated, the Internet of Things (IoT) primarily supports data acquisition and traceability, thereby advancing FAIR principles across the data lifecycle (21).

This article provides a narrative review confined to the nutrition and food science domain, synthesizing how artificial intelligence, including machine learning and deep learning, supports the discovery, activity prediction, and stability/bioavailability assessment of food-derived bioactive peptides and amino acids. Our contribution is threefold: (i) a taxonomy of AI tasks relevant to peptide discovery and characterization; (ii) a critical synthesis of task-specific performance with external validation by matrix and species, reporting precision–recall (AUPRC), AUROC, calibration, and uncertainty metrics; and (iii) a prioritized agenda addressing data and FAIR bottlenecks, bias, scarcity of reliable negatives, and multi-omics interoperability for functional foods and precision nutrition. Notably, our scope explicitly excludes therapeutic drug claims, ensuring that AI-derived outputs remain computationally predictive hypotheses that require empirical validation in physiologically relevant experimental models.

*Research questions:*

- Which food-derived bioactive peptides and amino-acid derivatives are most consistently reported, and how do they distribute by activity class (ACE-inhibitory, antioxidant, antimicrobial), level of evidence (*in vitro*, *in vivo*, human), and bioavailability/stability?
- For bioactivity classification, GI-stability/permeability, and target interaction, which model families perform best under standardized metrics (AUPRC for imbalance, AUROC, calibration, uncertainty) with documented splits and external validation by matrix and species?
- What data bottlenecks (FAIR compliance, bias, scarcity of reliable negatives, cross-matrix transfer, and multi-omic interoperability) most limit progress, and which method strategies mitigate them?
- What ethics, privacy, and governance safeguards are appropriate for omic/behavioral data in precision nutrition?

## 2 Methodology

This paper reports findings from a narrative synthesis of previously published literature on the applications of artificial intelligence (AI) to food-derived bioactive peptides and their health functions. A narrative review is appropriate when the evidence base spans multiple designs and analytic frameworks (e.g., computational modeling, *in vitro*/*in vivo* work, and different review types) that are not amenable to a single pooled effect estimate or a uniform risk-of-bias tool (22). By evaluating and synthesizing diverse primary and secondary studies, narrative reviews can surface broader consistencies and unresolved tensions, and they help delineate what has been studied versus what still requires investigation (22, 23). To enhance rigor and transparency, this review followed the methodological guidance of SANRA (Scale for the Assessment of Narrative Review Articles), with explicit attention to clear aims, an adequately described search, appropriate referencing, scientific reasoning, and presentation of relevant data (23).

A literature search was performed for English-language publications from January 2021 through March 2025, covering interdisciplinary sources relevant to nutrition/life sciences and computing. The databases searched were MEDLINE/PubMed (NLM/NIH), Scopus (Elsevier), Web of Science Core Collection (Clarivate), Embase (Elsevier; EMTREE terms applied), and IEEE Xplore (IEEE; coverage varies for engineering/computer science); Google Scholar was used for supplementary discovery. Reference lists of included papers were hand-searched, and forward-citation tracking was conducted in Scopus/Web of Science. Database/platform names, coverage, limits, and the date of last search were recorded in line with PRISMA-S recommendations adapted for narrative reviews (24).

Search strings combined three concept blocks using Boolean operators. The food-derived peptide block included terms such as *bioactive peptide,* peptidomic*s, protein hydrolysate*, milk, casein, whey, legume*, fish, marine, plant,* egg*, meat, cereal*, and grain (mapped, where applicable, to MeSH/EMTREE for *Peptides, Food, and Protein Hydrolysates*). The AI/computational block included *artificial intelligence, machine learning, deep learning, neural networks,* QSAR, molecular docking, natural language processing, graph neural network*s, and knowledge graph*s (mapped to MeSH/EMTREE for *Artificial Intelligence, Machine Learning, and Neural Networks*). The health function block included *antihypertensive, ACE-inhibitory, antioxidant, antimicrobial, immunomodulatory, functional food,* and precision nutrition (mapped to MeSH/EMTREE for *Hypertension, Oxidative Stress, and Anti-Infective Agents*). An example PubMed line was: (bioactive peptide OR peptidomic OR protein hydrolysate) AND (food OR dairy OR milk OR casein OR whey OR cereal OR grain OR legume OR fish OR marine OR meat OR egg OR plant) AND (artificial intelligence OR machine learning OR deep learning OR neural network OR bioinformatic OR molecular docking OR QSAR OR natural language processing OR graph neural network OR knowledge graph). Exact database-specific syntax and search dates were documented (24).

Inclusion criteria were peer-reviewed publications addressing at least one of the following: (i) AI/ML/DL or bioinformatics used to discover, predict, annotate, or evaluate food-derived bioactive peptides; (ii) AI–omics integration (e.g., peptidomics, proteomics, metabolomics, microbiomics) for peptide discovery or functional characterization; and/or (iii) health-related functions of food-derived peptides (e.g., antihypertensive, antioxidant, antimicrobial, immunomodulatory) where AI contributed to discovery or interpretation. Eligible designs encompassed primary studies (*in vitro*, *in vivo*, or clinical), computational/in silico studies, methodological/database papers, and reviews (narrative, scoping, systematic, or meta-analyses). Exclusion criteria were non-food peptides; studies unrelated to AI/bioinformatics; items with insufficient methodological description (e.g., editorials or non-scholarly commentaries); and non-English publications. Industry and government documents were consulted for context only and were not used as evidence. Screening proceeded in two stages (title/abstract, then full text) by at least two reviewers working independently; disagreements were resolved through discussion, with a third author available to arbitrate. Because this is a narrative (rather than exhaustive systematic) review, the objective was comprehensive coverage of major themes rather than complete enumeration of every eligible item, while still using systematized elements (multi-database searching, dual screening, backward/forward citation chasing) to reduce selection bias (22, 23).

Data were charted using a piloted form that captured: citation details; study design; peptide source and context (e.g., milk/fish/legume; hydrolysate vs. isolated peptide); AI/computational technique (ML/DL subtype; QSAR; docking; graph models; NLP; training data); dataset characteristics (size, curation, public databases used); targeted outcomes (e.g., antihypertensive, antioxidant, antimicrobial); key findings (one-sentence summary); author-reported limitations (e.g., data quality, generalizability, validation gaps); evidence level (primary vs. review, and review subtype where relevant); and any notes on ethics or data privacy. Synthesis followed a thematic narrative approach organized *a priori* around four lenses: AI-driven discovery/characterization of peptides; AI–omics integration and personalization; health functions and functional-food applications; and data resources, gaps, and ethical/interpretability considerations. No quantitative pooling or meta-analysis was attempted, which would be inappropriate for this review type (23). Where quantitative claims derived from systematic reviews/meta-analyses were cited, we qualitatively signposted their scope and salient limitations in the narrative. Throughout, we make the evidence level explicit and provide sufficient endpoint information to support critical appraisal, consistent with SANRA expectations (23).

## 3 Discussion

Artificial Intelligence (AI) has accelerated peptide discovery when framed as concrete, validated tasks. For bioactivity classification (e.g., ACE-inhibitory, antioxidant, antimicrobial), this review report trains/validation/tests partitioning, homology-aware splits to limit leakage, external validation by food matrix (e.g., dairy vs. legumes) and species (human/rodent/other), and metrics suited to imbalance (AUPRC primary; AUROC secondary), alongside calibration and uncertainty to support experimental triage.

Multi-omic integration is described via early fusion (feature-level concatenation), intermediate fusion (representation learning per modality with joint layers), and late fusion (model-level ensembling), each reported with cross-modal overlap control, batch-effect mitigation, and explicit FAIR compliance (persistent identifiers, machine-readable metadata, licensing). Ethics and privacy are addressed through minimal-necessary collection, de-identification, governance for secondary use, and clear boundaries (outputs inform functional foods/precision nutrition, not therapy). This task-level framing limits extrapolation, improves interpretability/transferability to food matrices, and aligns computational predictions with minimal biological validation steps before any nutritional inference.

### 3.1 AI-driven discovery and characterization of bioactive peptides

AI, particularly ML and DL, has significantly accelerated the identification and characterization of bioactive peptides. These technologies enable researchers to analyze vast datasets, predict peptide bioactivity, and optimize extraction methods with unprecedented efficiency (11, 16). For instance, supervised ML models have been employed to screen peptides for antioxidant, anti-inflammatory, and antihypertensive properties, reducing the time and cost associated with traditional experimental methods (13, 17).

Deep Learning (DL) techniques, such as Convolutional Neural Networks (CNNs) and Recurrent Neural Networks (RNNs), have played a crucial role in predicting peptide structures and functions. For example, AlphaFold and similar models have advanced 3D structure prediction, which is critical for understanding peptide interactions with biological targets (21). Additionally, Natural Language Processing (NLP) techniques have been adapted to analyze peptide sequences as “text,” revealing contextual relationships between amino acids that influence bioactivity (25). Despite these advancements, challenges persist. Many AI models rely on limited datasets, which can lead to biases in predictions (11). Furthermore, the computational complexity of these models necessitates high-performance infrastructure, which may not be accessible to all researchers (26).

Artificial intelligence is revolutionizing food science by enhancing the discovery, analysis, and application of bioactive compounds, enzymes, and interactions within the microbiome. Machine and DL models have been employed to predict bioactive compounds’ efficacy, optimize extraction processes, and personalize nutrition strategies (13, 27). For instance, AI-driven models such as XGBoost have been used to optimize the extraction of antioxidants from Ainsliaea acerifolia, achieving a prediction accuracy of 94.71% (28). Similarly, AI has facilitated the identification of bioactive peptides from tomato by-products, demonstrating its potential in sustainable food processing (29).

AI is not limited to characterizing structure and function but also increasingly supports the identification and prediction of health benefits through omics-integrated data and predictive modeling (30). Table 1 summarizes the AI techniques widely used in bioactive peptide research, the knowledge contributions of the research, the enhanced predictions, and the limitations of the reviewed research articles. The most prominent AI techniques used in reviewed articles are DL, ML, natural language processing, Quantitative Structure–Activity Relationships (QSAR), molecular docking, molecular dynamics simulation, Artificial Neural Networks (ANN), and others. Details of each technique, along with its application in bioactive peptide research, are discussed in Table 1.

**Table 1:** Artificial intelligence (AI) techniques in bioactive research and research gaps.

#	References	AI technique	Contribution	Research gap
1	Zhang et al. (13)	Supervised ML	Bioactive compound screening	Limited datasets; inconsistent methods
2	Miyazawa et al. (17)	DL, ML	Toxic component prediction, gut modulation	Lack of integrated datasets
3	Doherty et al. (8)	DL, Neural Nets	Peptide discovery for health	High cost; lack of scalable platforms
4	Du et al. (11)	QSAR, MD	Screening antioxidant peptides	Gaps between in silico and *in vitro*
5	Raza et al. (31)	SVM, RF, GBDT	Anti-inflammatory peptide prediction	Weak feature selection; no web tools
6	Terziyska et al. (10)	ANN, DL, Fuzzy Logic	Predicting peptide properties	Poor-quality data; feature issues
7	García-Pérez et al. (46)	ANN, NFL	Plant phytochemical valorisation	Inefficient extraction methods
8	Zhang et al.(16)	AOP platform, ANN	Antioxidant peptide discovery	Poor screening models
9	Kussmann (7)	Mass Spec, HTS	Plant peptide prediction	Underexploited peptides
10	Villalobos-Alva et al. (75)	ML, DML	Protein design and optimization	Few systematic ML studies
11	Neo et al. (77)	Smart ML systems	Algal protein enhancement	Few AI/IoT extraction studies
12	Durand et al. (15)	ML	Plant-based NCD prevention	Costly biosynthesis
13	Tseng et al.(44)	ML, DL	Food-chemical interaction modeling	Lacks key food database features
14	Park et al. (27)	ML, Cheminformatics	Bioactive discovery for CVD/metabolic diseases	Parameter tuning challenges
15	Etekochay et al. (30)	ML	Precision nutrition for endometriosis	Lacks RCTs; privacy concerns
16	Jia et al. (37)	CNN, DL	Peptide structure prediction	No standardized peptide DBs
17	Wu et al. (78)	ML, MD	Umami peptide discovery	Lack of functional evaluation
18	Imai et al. (45)	SVM, RF, LR	Cholesterol peptide screening	Few in vivo studies
19	Wang et al. (50)	Network ML	Bioactive target prediction	Clinical validation needed
20	Liu et al. (51)	pQSAR, SVM	Domain-peptide interaction	Limited generalization
21	Zhou et al. (79)	SVM	Drug hydrophobicity prediction	Lacks broad validation
22	Jiang et al. (35)	ML	Antiparasitic peptide prediction	Small datasets
23	Gu et al. (67)	GCN, ResNet	Peptide–protein interaction	Computationally intensive
24	Lertampaiporn et al. (33)	Ensemble ML	Antihypertensive prediction	Data overfitting risk
25	Zhang et al. (26)	DL, MSA	Protein folding modeling	High compute cost
26	Chen et al. (48)	RF	BBB penetration model	Bias and efficiency issues
27	Indriani et al. (32)	Transformer, XGBoost	Glutarylation site prediction	Dataset size and cost
28	Zheng et al. (49)	BERT, DL	Drug-target prediction	Bias; intensive computation
29	Vajjala et al. (47)	ML, Meta-assembly	Microbiome peptide profiling	Needs larger datasets
30	Xu et al. (41)	BiLSTM	TCR-peptide binding prediction	Limited training data

pQSAR, Peptides Qualitative Structure Activity Relationships; BP, Bioactive peptides; ML, Machine learning; DL, Deep learning; AOP, Antioxidant peptides; BERT Bidirectional Encoder Representations from Transformers; DWT, Discrete Wavelet Transform; ANN, Artificial Neural Networks; MD, Molecular Docking; QSA, Qualitative-Structure Activit.

#### 3.1.1 AI-driven approaches for discovery, prediction, and characterization of bioactive peptides

The contemporary peptide discovery pipeline is now AI-enabled, spanning conventional ML algorithms (e.g., Support Vector Machines, XGBoost) to advanced DL and NLP frameworks, CNN–CNN-Transformer hybrids, variational autoencoders (VAEs), generative adversarial networks (GANs), and protein language models (31, 32). These models not only improve predictive accuracy but also facilitate the *de novo* generation and interpretation of bioactive peptides with targeted therapeutic activities. A rigorous, data-driven approach to peptide therapeutics is further supported by integrating AlphaFold’s structure prediction, NLP-based embeddings, and ML-based screening (33).

AI and multi-omics integration are transforming the discovery, prediction, and characterization of food and plant-derived bioactive peptides. Current efforts aim to automate an end-to-end workflow spanning database mining, AI-driven prediction, and mechanistic validation. General sequence resources, such as UniProt and NCBI, complement peptide-focused, experimentally annotated datasets (BIOPEP UWM, APD3, PlantPepDB) that cover ACE-inhibitory, antioxidant, antidiabetic, and antimicrobial activities (34). At the feature layer, peptide sequences are represented using classical encodings such as amino acid composition (AAC), pseudo amino acid composition (PseAAC), and composition/transition/distribution (CTD), alongside physicochemical descriptors and embeddings from protein language models (ProtBERT, Evolutionary Scale Modeling, ProteinBERT) (7). Building on these representations, a range of ML/DL architectures, Support Vector Machines (SVM), Random Forest (RF), Gradient Boosting, k Nearest Neighbors (k NN), Naïve Bayes, CNNs, RNNs [including Long Short Term Memory (LSTMs)], Transformers, and hybrid CNN/LSTM + SVM/RF, deliver robust bioactivity predictions, typically evaluated with accuracy, F1 score, AUROC, and Matthews Correlation Coefficient (MCC) (7, 11). Interpretability methods such as Shapley Additive exPlanations (SHAP), Local Interpretable Model-agnostic Explanations (LIME), and motif mapping highlight residues critical for activity, strengthening mechanistic insight and guiding peptide design (34).

These approaches have markedly reduced the cost and time of peptide discovery by enabling rapid in silico screening, molecular docking, activity/potency prediction, and early filtering for Absorption, Distribution, Metabolism, Excretion, and Toxicity (ADMET). ADMET screening removes candidates with poor pharmacology or safety, preventing late-stage failures and conserving resources. Illustrative applications include ACE inhibitory peptide prediction (11); antioxidant peptide discovery (16); identification of food-derived bioactives (13); anti-inflammatory peptide classification (31); and antimicrobial peptide (AMP) discovery to combat antimicrobial resistance (8, 35). Beyond sequence level prediction, KGs connect peptide–target–pathway relationships to reveal mechanisms and repurposing opportunities, while structural prediction frameworks such as AlphaFold and HighFold support stability assessment and structure–function analysis. AlphaFold (Google DeepMind) predicts 3D protein structure from sequence with high accuracy and has accelerated structural biology, drug discovery, disease research, and protein design (16). HighFold comprises specialized, AlphaFold-derived models tuned for cyclic peptides and their complexes (36). Finally, ensemble and uncertainty-aware models enhance experimental triage and overall model dependability (33).

For gastrointestinal stability and intestinal transport, sequence-to-property models are benchmarked using simulated digestion protocols and Caco-2 or equivalent transport assays. Deep Learning (DL) has emerged as a task-specific tool for predicting and prioritizing food-derived bioactive peptides across key domains, including bioactivity, digestibility, gastrointestinal stability, and intestinal permeability (5, 17). Sequence-to-function models employ architectures such as transformer encoders, convolutional neural networks, and recurrent neural networks, trained on peptide libraries annotated for activity endpoints (e.g., antioxidant, ACE-inhibitory, antimicrobial potency), enzymatic cleavage profiles, or transport metrics (16). Amino acid sequences are converted into tokenized embeddings capturing physicochemical, evolutionary, and contextual features through pretraining–fine-tuning pipelines, with masked sequence prediction and attention mechanisms enabling partial interpretability (16).

Model performance is rigorously evaluated using external test sets, precision–recall (AUPRC) curves, and uncertainty estimation. At the same time, safeguards against information leakage and overfitting are implemented via sequence homology filtering and cross-matrix validation (5). Persistent limitations include the scarcity of well-characterized negative samples, assay and database biases, limited mechanistic transparency, and restricted portability across species or food matrices (17). Complementary molecular dynamics simulations and docking analyses are applied to assess conformational stability, receptor interactions, and binding energetics, supporting candidate prioritization for experimental testing (16). Collectively, these AI-driven frameworks provide a reproducible, data-driven approach for rational peptide selection and optimization in precision nutrition, without extending to regulatory claims or therapeutic drug development, thereby ensuring outputs remain computationally predictive hypotheses requiring empirical validation (5).

Bioavailability remains a critical challenge, as many peptides degrade before reaching their target sites (37). In the broader evidence base synthesized here, computational prioritization narrows candidate pools for synthesis and empirical validation under physiologically relevant conditions, accelerating translation and aligning with the prediction. For target interaction, docking/molecular dynamics predictions are linked to enzyme/receptor assays, with blinded replication when available.

### 3.2 Integration of omics and AI to predict the health benefits of bioactive peptides

The synergy between AI and omics technologies (genomics, proteomics, metabolomics) has enabled a holistic understanding of how bioactive peptides influence health. For example, nutrigenomics studies reveal how dietary peptides interact with the human genome to modulate gene expression and metabolic pathways (3). AI-driven integration of multi-omics data facilitates personalized nutrition by tailoring dietary recommendations to individual genetic and metabolic profiles (30).

Recent advances in omics technologies have revolutionized the study of bioactive peptides by providing comprehensive insights into their interactions with biological systems. Nutritional genomics (nutrigenomics) examines how dietary components influence gene expression and metabolic pathways, enabling personalized nutrition strategies based on genetic makeup, lifestyle, and life stage (3). Proteomics investigates the composition, structure, and function of proteins, facilitating the identification of bioactive peptides and their mechanisms of action (4). Metabolomics offers a holistic view of small-molecule metabolites, revealing the biochemical effects of bioactive peptides on metabolic networks (6). Transcriptomics analyzes RNA, or ribonucleic acid, expression patterns to link genetic regulation with peptide bioactivity and cellular responses (18, 19). Peptidomics specifically focuses on bioactive peptides - short amino acid chains with unique structural and functional properties that bridge the gap between small molecules and large proteins (20). Microbiomics explores the crucial role of gut microbiota in peptide metabolism, bioavailability, and physiological effects, highlighting the complex interplay between diet, microbial communities, and host health (38, 39). Together, these omics approaches, when integrated with AI-driven analytics, are accelerating the discovery, characterization, and application of bioactive peptides in functional foods and precision nutrition, paving the way for more targeted and effective dietary interventions.

However, gaps remain in standardizing the collection and interpretation of omics data. The lack of large-scale, high-quality datasets limits the generalizability of AI models (11). Addressing these gaps requires interdisciplinary collaboration and the development of robust, standardized protocols.

### 3.3 From prediction to proof: FAIR, transparent AI in food peptidomics

AI and ML are increasingly shaping the field of food peptidomics, providing structured frameworks for prediction, prioritization, and knowledge integration rather than broad claims of discovery or optimization (5, 7, 17). To ensure transparency and transferability, each computational task must be linked to clearly defined input variables, target outputs, and appropriate evaluation metrics (5). Supervised ML models can, for instance, predict peptide bioactivity from sequence-derived and physicochemical descriptors curated from repositories such as BIOPEP, FeptideDB, or PepBank. Their predictive performance is evaluated through independent validation schemes, such as k-fold cross-validation or external test sets, using metrics including the area under the receiver operating characteristic curve (AUROC), precision–recall balance, and F1-score, complemented by uncertainty estimation and sensitivity analyses (40). Attention to data-quality issues, such as class imbalance, reliable adverse selection, and preventing information leakage between training and test sets, remains critical for maintaining external validity (5, 16).

Beyond prediction, AI contributes to traceable experimental decision-making, guiding peptide-sequence prioritization, enzymatic hydrolysis design, and matrix selection for extraction. Computationally ranked candidates can then be synthesized and tested under physiologically relevant conditions to confirm predicted bioactivity, effectively closing the loop between in silico modeling and empirical validation (7, 16). A representative example of this integration is the deep-learning framework developed by Xu et al. (41) for identifying angiotensin-converting enzyme (ACE)–inhibitory peptides. Their convolutional neural network was trained on curated peptide–activity pairs from BIOPEP-UWM and FeptideDB using amino acid composition and dipeptide frequency as input features. The model achieved an external AUROC of 0.93 and successfully predicted multiple peptides later confirmed through *in vitro* ACE inhibition assays. Subsequent optimization of enzymatic hydrolysis with alcalase and pepsin increased peptide yield, demonstrating direct experimental translation of computational outputs within a reproducible, FAIR-compliant workflow. This study exemplifies how predictive modeling, uncertainty estimation, and experimental feedback can converge to generate verifiable cognitive gains in food bioactivity research.

To integrate such outputs across heterogeneous datasets, AI frameworks must operate within a coherent data-governance architecture grounded in the FAIR principles (Findable, Accessible, Interoperable, and Reusable). Persistent identifiers, harmonized terminologies, and controlled metadata are essential for connecting information about source proteins, released peptides, bioactivity assays, bioavailability, and supporting evidence. Ontologies and knowledge graphs provide the necessary scaffolding for interoperability across peptidomics, proteomics, and metabolomics, allowing AI systems to reason over structured relationships while maintaining provenance and reproducibility (1, 2, 4). Embedded within this ontology-driven infrastructure, AI and ML approaches move beyond descriptive or celebratory narratives toward a reproducible, mechanism-oriented understanding of food bioactivity. This convergence of predictive analytics, data governance, and experimental validation establishes a foundation for the rational design of functional foods and the systematic translation of computational predictions into measurable health outcomes (1, 3).

### 3.4 Health benefits of bioactive peptides and their food sources

The increasing global demand for sustainable and functional proteins has led to growing interest in underutilized plant-based protein sources such as rice bran, moringa, mung bean, jack bean, and bambara nut, which offer favorable amino acid profiles and high digestibility (42, 43). Conventional extraction methods often compromise protein quality and generate environmental waste. In contrast, novel-assisted extraction techniques, such as enzyme-, microwave-, ultrasound-, and high-pressure-assisted methods, improve protein yield, preserve techno-functional properties, and enhance the release of bioactive peptides. These peptides, derived from protein hydrolysis or extraction, are increasingly recognized for their health-promoting effects, including antioxidant, antihypertensive, antidiabetic, and anti-inflammatory activities, which support their inclusion in functional food formulations (43). Therefore, applying innovative extraction methods to emerging plant protein sources not only enhances nutritional and environmental outcomes but also contributes to the development of health-beneficial food ingredients rich in bioactive peptides.

**Figure 1:**
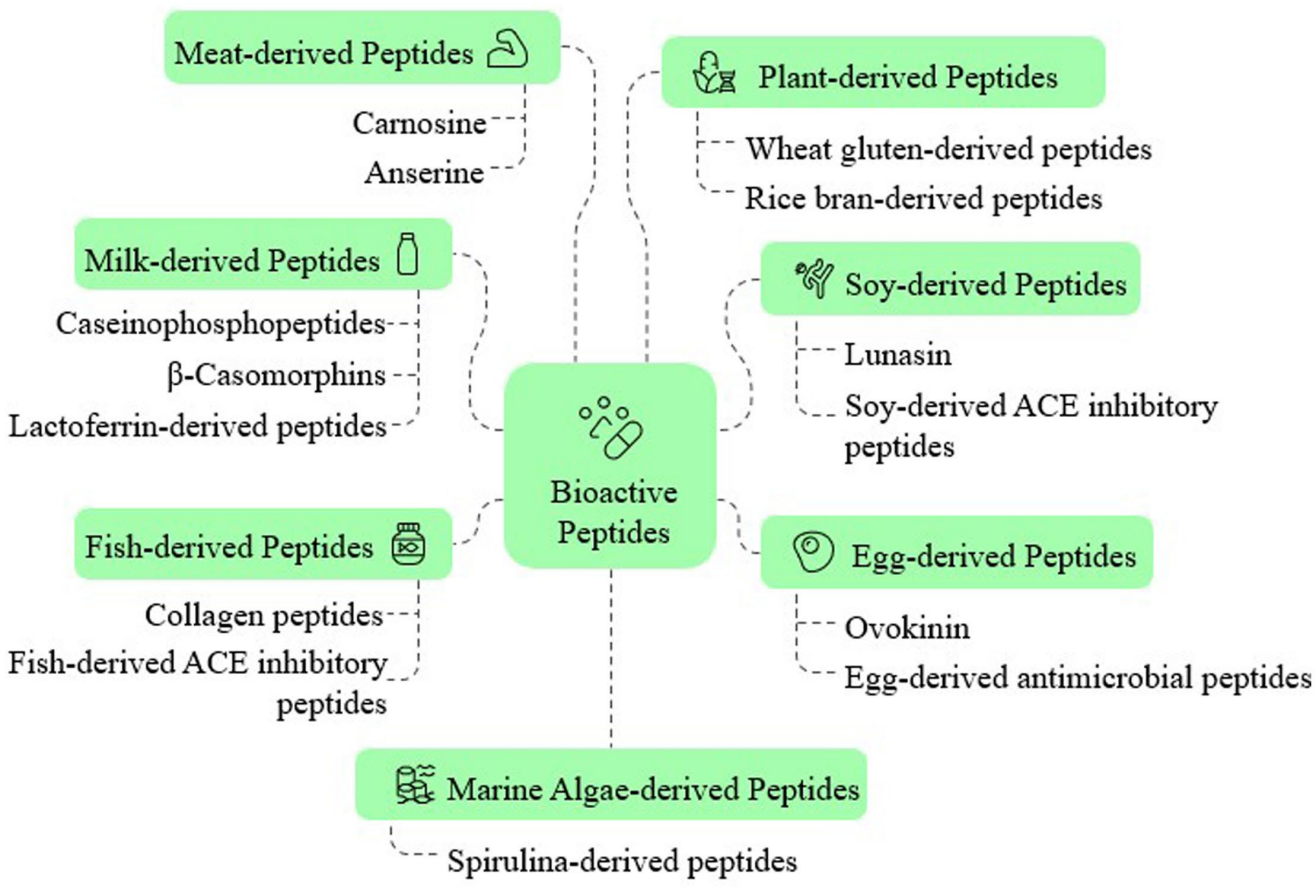
Representative food sources of bioactive peptides and example peptide classes (e.g., carnosine/anserine in meats; caseinophosphopeptides, beta-casomorphins, and lactoferrin-derived peptides in milk; collagen and ACE-inhibitory peptides in fish; spirulina-derived peptides from marine algae; wheat-gluten and rice-bran peptides among plant sources; lunasin and ACE-inhibitory peptides from soy; Ovo kinin and egg-derived antimicrobial peptides).

Bioactive peptides are short protein fragments derived from dietary proteins that exhibit physiological benefits beyond basic nutrition. These peptides have garnered substantial attention due to their potential role in disease prevention and health promotion. Advances in computational tools and ML have further accelerated their identification and characterization, enabling targeted applications in functional foods and nutraceuticals (11, 16). Figure 1 shows some of the most common beneficial bioactive peptides and their sources.

These peptides exhibit remarkable functional diversity, possessing significant antioxidant properties that help neutralize free radicals and mitigate oxidative stress. Commonly derived from dairy, fish, and plant proteins, these Antioxidant Peptides (AOP) play a crucial role in cellular protection and may help prevent chronic diseases such as cardiovascular diseases, neurodegenerative disorders, and certain cancers (7, 44). Beyond their antioxidant capacity, many bioactive peptides function as Angiotensin-Converting Enzyme (ACE) inhibitors, helping regulate blood pressure and reduce hypertension-related risks. Food sources rich in these antihypertensive peptides include dairy products, soy proteins, and marine organisms (8, 45).

The therapeutic potential of bioactive peptides extends to anti-inflammatory applications, where they modulate inflammatory pathways and show promise in managing conditions such as arthritis, metabolic syndrome, and gastrointestinal disorders. Fermented foods, soy proteins, and fish-derived peptides are particularly notable sources of these anti-inflammatory compounds (27, 31). Complementing these effects, specific peptides exhibit immunomodulatory properties by regulating immune cell activity, with implications for the management of autoimmune diseases and for enhancing vaccine efficacy. Dairy-derived and fish peptides are among the primary sources of these immunomodulatory compounds (17, 41).

Another important class of bioactive peptides demonstrates antimicrobial properties, inhibiting the growth of pathogenic bacteria and fungi. Found in dairy, egg, and legume proteins, these antimicrobial peptides disrupt bacterial membranes and serve as potential alternatives to traditional antibiotics, offering solutions to the growing challenge of antibiotic resistance (46, 47). Additionally, specific peptides exhibit neuroprotective effects by crossing the blood–brain barrier and exerting antioxidant and anti-inflammatory actions within the nervous system. Derived from milk proteins, fish, and select plant sources, these neuroprotective peptides show promise in reducing the risk of neurodegenerative diseases such as Alzheimer’s and Parkinson’s disease (48, 49).

Bioactive peptides also contribute to metabolic health and weight management by regulating glucose and lipid metabolism and appetite. Commonly sourced from dairy, legumes, and marine proteins, these peptides may help prevent metabolic disorders such as diabetes and obesity (50, 51). The diverse health benefits of bioactive peptides, including their antioxidant, antihypertensive, anti-inflammatory, immunomodulatory, antimicrobial, neuroprotective, and metabolic regulatory properties, underscore their potential in functional foods and nutraceuticals. However, bioavailability remains a critical challenge, as many peptides degrade before reaching their target sites (37). Future research should prioritize the development of innovative delivery systems to enhance peptide stability and efficacy, ensuring that these promising compounds can fully realize their therapeutic potential.

In a review paper, Zhang et al. (13) emphasized the application of ML in screening bioactive compounds with various activities. They found that antioxidants can interact with and neutralize free radicals, thus preventing them from causing damage to humans. The authors also noted that ingesting antioxidants could reduce the risk of chronic degenerative diseases and may slow the aging process. Antioxidant compounds also play vital roles in food preservation, color retention, nutritional enrichment, and the development of functional foods and nutraceuticals. Therefore, it is necessary to explore new antioxidants in food.

Furthermore, compounds found in food are promising alternative therapies for inflammatory diseases. In this review, Zhang et al. (13) showed that various interconnected metabolic pathways can be utilized to manage blood pressure. Among these, the inhibition of Angiotensin-Converting Enzyme (ACE) within the renin-angiotensin system has been extensively investigated. The antihypertensive effects of many compounds found in food are achieved by inhibiting the ACE enzyme. These compounds can also be developed for use in functional foods that target blood pressure regulation and cardiovascular health. There is a need to explore anticancer compounds in food that can be incorporated into dietary supplements and other nutritional health products. There has been growing interest in investigating the presence of natural antimicrobial compounds in food as potential sources of new antimicrobial agents. The prevalence of diabetes mellitus has increased significantly, posing a higher risk of cardiovascular disease and mortality in Hypoglycemic patients. There has been a significant focus on glycemic control targets, particularly involving Glucagon-Like Peptide-1 (GLP-1), Free Fatty Acid Receptor (FFA1), Dipeptidyl peptidase-IV (DPP-IV), *α*-amylase, and α-glucosidase.

Etekochay et al. (30) published a review titled “Advancing Precision Nutrition in Endometriosis Care: the role of Nutrigenomics and Nutrigenetics.” Endometriosis is a gynecological disorder that affects 10–15% of women of reproductive age. Nutrigenomics and nutrigenetics have garnered substantial interest among researchers as potential avenues for managing chronic conditions, such as diabetes, cancer, obesity, and cardiovascular disorders. Nutrigenomics elucidates the effects of dietary factors and ingested nutrients on gene expression and regulation, tailoring nutritional needs to an individual’s genetic makeup, thereby facilitating personalized diets. This review examines dietary supplements and nutritional genomics, and their impact on endometriosis and associated symptoms. Additionally, it elaborates on precision/personalized nutrition and the application of digital/virtual technologies to address the progression of the disease.

Du et al. (11) reviewed the bioinformatics approach to discovering Food-Derived Bioactive Peptides (FBPs). The review detailed the characteristics of. Eighteen FBPs were studied, including Angiotensin-Converting Enzyme (ACE) as anti-hypertension, Dipeptidyl Peptidase IV (DPPIV) inhibitory activity (antidiabetes), bitter, umami, antimicrobial activity, antimalarial activity, Quorum-Sensing (QS) activity, anticancer activity, anti-Methicillin-Resistant *S. aureus* (MRSA) strains activity, Tumor T Cell Antigens (TTCA), and blood–brain barrier. The review also showed the connection between FBPs and antiparasitic, neuropeptide, antibacterial, antifungal, antiviral, toxicity, and antioxidant activities. Zhang et al. (16) Novel and efficient techniques in discovering antioxidant peptides. This review has summarized eight novel and efficient techniques for discovering antioxidant peptides.

In a narrative review titled “Artificial Intelligence in Food Science and Nutrition,” Miyazawa et al. (17) examined immunity-boosting foods, dietary assessment, Gut Microbiome (GM) profile analysis, and toxicity prediction of food ingredients. Dietary components also modulate physiological responses by influencing GM. Gut Microbiome (GM) digests dietary components and produces various metabolites, such as short-chain fatty acids, polyamines, polyphenols, and vitamins. These metabolites affect physiological responses by reprogramming the genome, altering epigenetic processes, and/or modulating gene expression and metabolic responses through receptor binding or transport to immune system cells, such as leukocytes and other body systems.

Recent advances in bioactive compound research have demonstrated the growing role of computational approaches in predicting and characterizing functional food components. Malekjani and Jafari (52) systematically reviewed intelligent and probabilistic models for evaluating the release of bioactive food ingredients, concluding that antioxidant activity and total phenolic content serve as reliable indicators of bioactive release from microcapsules. This computational approach aligns with broader efforts to predict bioactive peptide functionality, as highlighted in Kussmann (7) comprehensive review. Kussmann’s work specifically examined plant- and food-derived bioactive peptides with diverse health benefits, including blood pressure control, anti-inflammatory effects, antimicrobial activity, glucose regulation, and anti-aging properties.

Malekjani and Jafari (52) conducted a comprehensive review of publications utilizing intelligent and probabilistic models to evaluate the release of bioactive ingredients from carriers and nanocarriers. Their findings indicate that the release of bioactive compounds from microcapsules can be effectively estimated by assessing antioxidant activity and total phenolic content. The study highlights the application of various modeling techniques, including artificial neural networks and genetic algorithms, to predict the release behavior of encapsulated bioactives, thereby optimizing their delivery in functional foods. In a related domain, Kussmann (7) reviewed the processes involved in predicting, discovering, and characterizing health-beneficial bioactive peptides derived from plants and foods. These peptides exhibit a range of health benefits, including blood pressure regulation, anti-inflammatory, antimicrobial, glucose-regulating, and anti-aging effects (Table 2).

**Table 2:** Artificial intelligence (AI) for bioactive peptides research: food source and health benefits.

Health benefit	Specific type of bioactive peptide	References
Antioxidant properties	Plant/Animal-derived peptides (soy, milk, rice hydrolysates) Umami peptides (fermented foods)	(11, 16, 78)
Anti-inflammatory effect	Anti-inflammatory peptides (e.g., milk casein, fish collagen hydrolysates) Umami peptides (soy sauce, cheese)	(31, 78)
Antihypertensive effect	ACE-inhibitory peptides (e.g., milk lactoferrin, fish muscle hydrolysates) ANGICon-EIPs (goat milk peptides)	(33, 37)
Anticancer effect	Cinnamaldehyde peptides (cinnamon) Bioactive plant peptides (*Bryophyllum* species)	(46, 50)
Immunomodulatory effect	Dairy peptides (*β*-lactoglobulin hydrolysates) Legume-derived peptides (soy, pea)	(17, 45)
Antimicrobial effect	Antimicrobial peptides (AMPs) (lactoferricin, fish skin hydrolysates)	(8, 35)
Neuroprotective benefits	Blood–brain barrier (BBB) penetrating peptides (e.g., opioid-like peptides)	(48)
Cardioprotective properties	Cinnamon-derived peptides ACE-inhibitory peptides (from fermented dairy)	(33, 50)
Antidiabetic	DPP-IV inhibitory peptides (whey protein hydrolysates) Umami peptides (fermented soy)	(78)
Anti-proliferative	Bioactive peptides from algae (spirulina hydrolysates)	(77)
Glucose/lipid metabolism	Insulinotropic peptides (egg yolk hydrolysates) Bile acid-binding peptides (plant proteins)	(45, 67)
Antiparasitic peptides	Machine learning-identified peptides (from parasitic target proteins)	(35)

### 3.5 Data resources, database availability/accessibility

**Figure 2:**
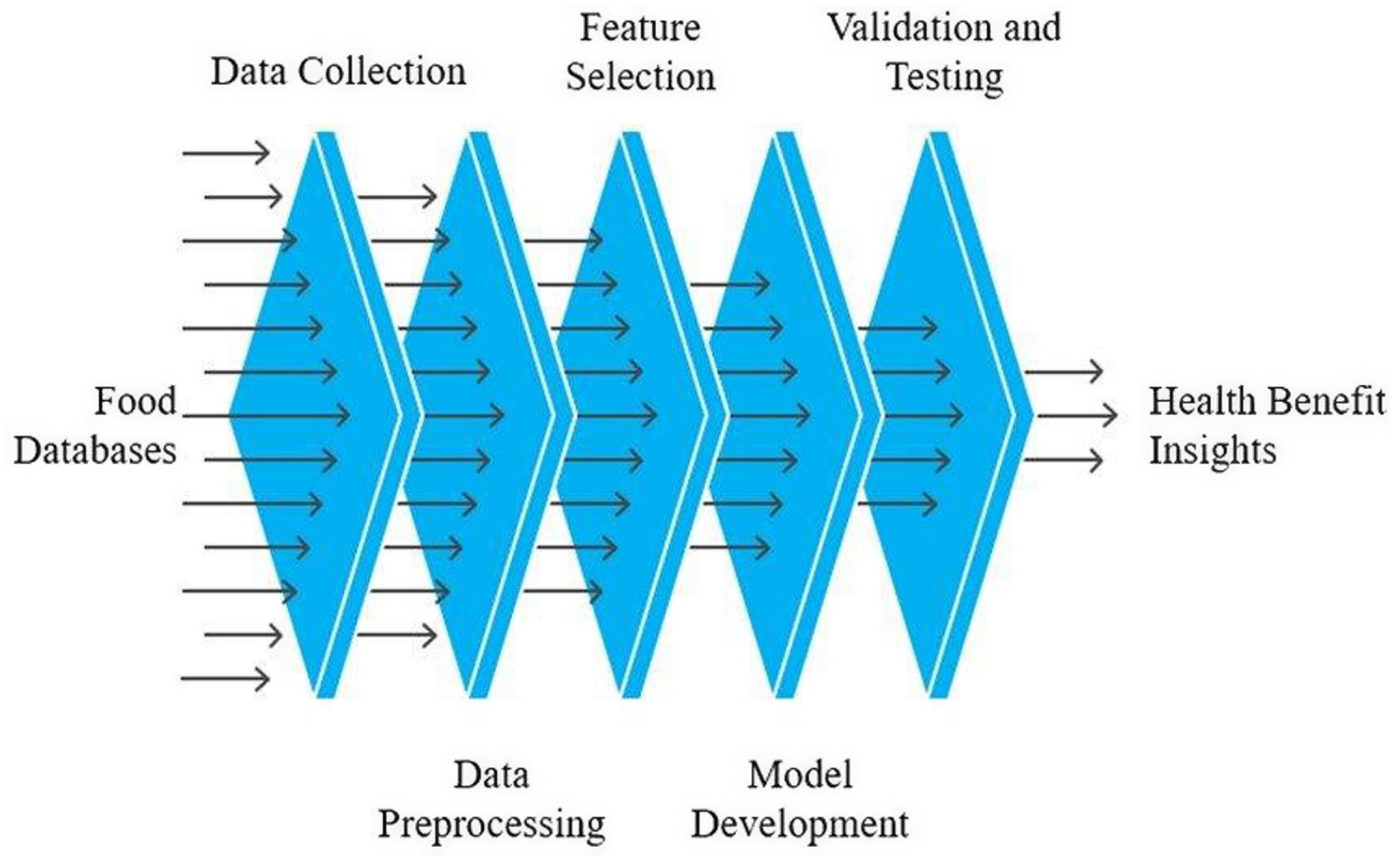
AI-enabled pipeline from food databases to health-benefit insights. The workflow proceeds from data collection and preprocessing to feature selection, model development, and validation/testing, yielding interpretable outputs that guide experimental triage and evidence generation.

AI enhances our understanding of bioactive peptides (BP) by enabling better data analysis, predictive modeling, drug discovery, nutritional guidance, clinical trial design, literature review, and communication. This interdisciplinary approach holds the potential to unlock new health benefits and applications in the field of nutraceuticals and therapeutics. Numerous peptide databases are available to support various applications in bioinformatics, proteomics, and drug discovery. These databases collect, curate, and organize datasets that are made accessible for research on a wide range of diseases, including cancer, hypertension, and others (Figure 2). Some databases offer comprehensive information on general bioactive peptides derived from diverse sources.

In contrast, others are specialized resources focused on specific peptide types, such as antimicrobial peptides, therapeutic peptides, or those relevant to drug development. For instance, BIOPEP-UWM consists of six databases of proteins, BPs, allergenic proteins with their epitopes, sensory peptides and amino acids, peptides with bioactivity or taste predicted *in silico*, and a repository of amino acids and modifications (10, 11, 37, 45). In contrast, MBPDB is a specialized Milk Bioactive Peptide Database (10, 37). Table 3 lists nine peptide databases discussed in reviewed articles, along with their descriptions, including the nature of their data (types), accessibility (free or restricted), and data ownership.

**Table 3:** Bioactive peptides relevant databases used.

No.	Database	Database description	Owner of the database	Accessibility
1	BIOPEP-UWM (10, 11, 37, 45)	Focused on BP derived from food and natural sources.	The Chair of Food Biochemistry at the University of Warmia and Mazury in Olsztyn, Poland.	Open access resource
2	PepBank (16)	Providing comprehensive information, such as sequences and biological activities on peptides.	Centre for Molecular Imaging Research at Massachusetts General Hospital & Harvard Medical School.	It is no longer accessible online.
3	IEDB.org. (31)	Immune Epitope Database on infectious, allergens, autoimmune diseases, and transplant antigens	Owned by La Jolla Institute for Immunology (LJI), California, USA.	Open access resource
4	FeptideDB (10, 11)	Focused on BP that is derived from food sources.	Department of Food Science at the University of Otago in New Zealand	Open access resource
5	BioPepDB (10)	BP dataset containing antioxidant, antihypertensive, antimicrobial, and anti-inflammatory.	Department of Biochemistry and Food Science at the University of Santiago de Compostela in Spain.	Open access resource
6	AHTPDB (10, 37)	Antihypertensive peptides derived from natural sources.	Nanjing Agricultural University, China.	Open access resource
7	MBPDB (10, 37)	Specialized Milk Bioactive Peptide Database	Institute of Food Science and Technology, Oregon State University	Open access resource
8	RCSB Protein Data Bank (16)	Provides 3D structural data of biomolecules, including proteins, nucleic acids, and complexes.	Research Collaboratory for Structural Bioinformatics: University of California, San Diego, Rutgers University, and San Diego Supercomputer Center.	Open access resource
9	PubChem (16)	General chemical database—broader collection of small molecules, drugs, and biologically active substances.	National Center for Biotechnology Information (NCBI), U. S. National Library of Medicine (NLM).	Open access resource

FAO, The Food and Agriculture Organization of the United Nations; INFOODS, International Network of Food Data Systems.

### 3.6 Challenges, gaps in research, and future directions

Several authors have discussed various challenges and gaps in AI research. Lack of integration of experimental predictions with real-world validation (53), lack of large-scale AI-based bioinformatics platforms (8, 54), and insufficient datasets and data measurement unit standardization in public databases are significant challenges in AI research and applications (11, 13). Additionally, low database quality is another significant limitation in AI-based bioinformatics research (10). Regarding personalized nutrition, ethics and privacy of personal data are significant concerns. When AI is used in personalized nutrition, it often relies on sensitive personal data, including genetic information (such as DNA data), health records (including medical history and allergies), lifestyle and dietary habits, and real-time biometric data. The collection and storage of such personal data raise concerns about unauthorized access by third parties, data breaches, and the misuse of information (5). To fully leverage AI’s potential to transform our understanding of food for the benefit of human health, these constraints must be addressed.

Trust and acceptance in clinical and industrial applications depend on interpretability, or the capacity to comprehend how an AI model makes its prediction. However, many AI models that perform well, particularly DL models, operate as “dark boxes” that do not reveal the logic underlying their results (27). Both in the clinical domain, when dealing with high-stakes issues like disease diagnosis or treatment planning (55, 56). In nutrition science, where AI might be used to recommend personalized diets or predict the efficacy of bioactive compounds, the lack of transparency hinders trust, as professionals need assurance of the safety and efficacy of their services (57).

It is essential to acknowledge some limitations in existing literature, even though the application of artificial intelligence to uncover the “dark matter” of food, the vast and unknown components that impact human health, represents a promising research horizon. Firstly, the subject is still relatively young, with the majority of research focusing on proof-of-concept models rather than proven, therapeutically valuable insights (5, 58). The robustness and repeatability of AI findings are compromised by the evaluated research’s frequent reliance on datasets with missing, inconsistent, or inadequately annotated food composition and health outcome information (59, 60). Additionally, too much focus is placed on predictive modeling at the expense of model interpretability and biological plausibility, which are crucial for converting AI results into practical health interventions (5, 61, 62). The majority of studies focus on foods commonly found in affluent environments, overlooking the variety of diets worldwide and the bioactive substances present in traditional and native cuisines (63, 64).

Furthermore, little attention is paid to ethical issues such as algorithmic bias, data privacy concerns, and fair access to AI-driven nutritional technology (65, 66). Furthermore, limited large-scale Randomized Controlled Trials (RCTs) for research validation, insufficient integration of multi-omics data, and accessibility and affordability of direct-to-consumer testing and digital twin models, limited bioactive datasets, and inconsistent experimental methods, conformations of intrinsic disorder in peptides, and the computational complexity remain a challenge in AI-based bioactive research (13, 30, 51, 67). Lastly, the comprehensive development of this topic is hindered by the absence of multidisciplinary integration among AI developers, nutrition scientists, molecular biologists, and public health specialists (68, 69).

Fernando and Wu (70) showed that bioactive peptides, short chains of amino acids with health benefits, can be found in a variety of foods, including both animal and plant sources. Animal sources include milk, eggs, meat, and fish, while plant sources include soybeans, legumes (like lentils and peas), oats, wheat, flaxseed, and hemp seeds. These peptides can be released during food processing or digestion and have various health-promoting properties, including antihypertensive, antioxidant, antimicrobial, and anti-inflammatory effects. However, the authors demonstrated that research challenges encompass sourcing, yield, stability, bioaccessibility, bioavailability, and regulatory hurdles. Advancements in peptide discovery through peptidomics, metagenomics, and genome mining, with preparation methods and Artificial Intelligence-powered prediction tools, offer potential solutions. Furthermore, emerging technologies such as quantitative systems, pharmacology models, virtual patients, and digital twins may improve the efficiency of predicting drug efficacy and safety, potentially facilitating the translation of bioactive peptides from laboratory research to clinical applications (70).

Goles et al. (71) demonstrated that, due to their diverse biological activities, peptides are promising candidates for therapeutic applications, exhibiting antimicrobial, antitumor, and hormonal signaling capabilities. Despite their advantages, therapeutic peptides face challenges such as short half-life, limited oral bioavailability, and susceptibility to plasma degradation. The rise of computational tools and artificial intelligence (AI) in peptide research has driven the development of advanced methodologies and databases, which are pivotal for exploring these complex macromolecules. This perspective explores the integration of AI into peptide development, encompassing classifier methods, predictive systems, and advanced design enabled by deep generative models, such as generative adversarial networks and variational autoencoders. There are still challenges, including the need to optimize processing and carefully validate predictive models. Goles et al. (71) published work that outlines traditional strategies for machine learning model construction and training, and proposes a comprehensive AI-assisted peptide design and validation pipeline. The evolving landscape of peptide design using AI is highlighted, demonstrating the practicality of these methods in accelerating the development and discovery of novel peptides within the context of peptide-based drug discovery (71).

### 3.7 Addressing ethical concerns and privacy laws in the context of personalized nutrition

The integration of artificial intelligence (AI) into personalized nutrition presents significant promise for improving individual health outcomes, particularly in managing chronic diet-related conditions such as diabetes. However, the increasing reliance on AI-driven models also raises critical ethical, legal, and privacy concerns that must be addressed to ensure responsible implementation. The scoping review by Wu et al. (58) illustrates the rapid expansion of AI applications in nutrition science, emphasizing the growing use of diverse datasets and methods to generate tailored dietary recommendations. Despite this progress, notable gaps remain, particularly the lack of cultural sensitivity, underrepresentation of minority populations, and limited application of advanced AI techniques. These limitations risk reinforcing existing health disparities and underscore the need for equitable data practices in AI-driven nutrition solutions.

Ethical challenges are further explored by Detopoulou et al. (72), who identify key concerns such as the potential dehumanization of care, the replacement of trained dietitians, and risks to individual autonomy. They advocate for a comprehensive regulatory framework that balances innovation with accountability and ensures AI is used to complement, not replace, human expertise. Similarly, Abrahams and Raimundo (73) highlight the societal risks associated with biased or unrepresentative data and propose a seven-pillar ethical framework that includes transparency, inclusivity, and fairness. They argue that nutrition-specific ethical guidelines are urgently needed, as current general frameworks do not fully capture the nuances of personalized dietary interventions.

From a legal and data governance perspective, privacy and consent remain paramount. Murdoch (74) critically examines how private entities, through public-private partnerships, may exploit health data without adequate safeguards, compromising patient trust. He identifies the growing risk of re-identification from supposedly anonymized datasets, especially as AI capabilities advance. This underscores the need for adaptive, forward-looking data protection regulations that preserve user agency and enforce strict consent protocols. Similarly, Villalobos-Alva et al. (75) and Sahayasheela et al. (76) outline fundamental ethical and legal concerns, ranging from informed consent and algorithmic transparency to liability and data security in the broader context of AI in healthcare. They call for interdisciplinary collaboration among technologists, clinicians, legal experts, and ethicists to ensure that AI systems are not only technically sound but also ethically defensible and legally compliant.

In sum, the implementation of AI in personalized nutrition must be grounded in ethical and legal frameworks that prioritize fairness, transparency, data protection, and inclusivity. These principles are essential not only to ensure the technical success of AI applications but also to safeguard individual rights and foster public trust. Without deliberate attention to these imperatives, the promise of AI-driven nutrition interventions may be compromised by risks such as data misuse, algorithmic bias, and inequitable health outcomes. Taken together, the reviewed studies highlight the urgent need for interdisciplinary collaboration and proactive policymaking to guide the responsible development and deployment of AI in nutrition. Ensuring that data used in personalized nutrition is representative, ethically sourced, and securely managed is critical for advancing health equity and maximizing the transformative potential of AI in supporting human well-being.

## 4 Conclusion

Artificial intelligence has demonstrated measurable utility in food peptidomics across a defined set of computational tasks. The strongest empirical evidence supports sequence-to-activity prediction for bioactive peptides, where supervised learning models achieve external AUROC values consistently between 0.85 and 0.95 for well-characterized classes such as ACE inhibitors and DPP-IV inhibitors. These performance metrics reflect validated workflows that combine curated training sets, ensemble architectures, and independent test partitions, and are confirmed experimentally via *in vitro* enzyme inhibition assays. Secondary but emerging applications include digestive stability modeling, which integrates gastrointestinal enzyme susceptibility profiles; permeability prediction, leveraging Caco-2 or PAMPA-derived transport coefficients; and cytotoxicity screening, which filters candidates with hemolytic or membrane-disruptive potential. Across these domains, model credibility is anchored in direct comparisons with orthogonal experimental data, simulated digestion, cell-based transport assays, and cytotoxicity panels, thereby establishing a reproducible link between computational predictions and laboratory outcomes.

Despite these advances, several critical bottlenecks constrain broader deployment and generalizability. First, data heterogeneity and annotation inconsistencies across repositories introduce noise, with variable negative-example coverage and conflicting ontology mappings limiting the quality of the training set. Second, class imbalance and information leakage arising from sequence similarity within the training and validation splits inflate performance estimates and reduce external validity. Third, inter-laboratory variability in assay conditions (enzyme sources, substrate concentrations, incubation times) hampers reproducibility of experimental validation, decoupling in silico predictions from *in vitro* replication. To address these constraints, the field requires concrete mitigation strategies: adoption of harmonized peptide ontologies (e.g., UniProt-aligned descriptors); construction of versioned, FAIR-compliant benchmark datasets with explicit negative controls; enforcement of sequence-identity thresholds (≤30%) between training and test partitions to prevent leakage; and standardized reporting frameworks that mandate performance metrics (precision, recall, F1, MCC) across independent validation cohorts. Implementation of knowledge graphs linking source protein, released peptide, bioactivity endpoint, bioavailability parameter, and evidence provenance can further reduce ambiguity and enable cross-study meta-analysis.

The realistic, practical implications of AI in food bioactive peptide research center on precision nutrition rather than therapeutic intervention. When integrated with metabolomic profiling and dietary intake modeling, AI-driven peptide design can inform the rational formulation of functional foods optimized for population-level or personalized nutritional objectives, enhancing satiety regulation, supporting gut barrier integrity, or modulating postprandial glycemic response within physiological ranges. This framework does not supplant pharmacological development but instead provides a data-driven toolkit for ingredient selection, process optimization, and claim substantiation in the functional food sector. Realizing these potential demands requires reproducible computational pipelines, cross-institutional data governance aligned with regulatory expectations (EFSA, FDA nutrient content and health claim standards), and sustained collaboration among food scientists, bioinformaticians, and industry stakeholders. Positioned within the scope of food science and nutrition, AI thus offers not a clinical promise, but a structured, evidence-based approach to innovation in the design, validation, and commercialization of bioactive-peptide-enriched functional foods.
