# Supplementary material for: Can artificial intelligence uncover the bioactive peptides’ benefits for human health and knowledge? A narrative review
Source: Front Nutr. 2025 Dec 15;12:1698147. doi: 10.3389/fnut.2025.1698147 (PMC12746658; doi:10.3389/fnut.2025.1698147)
Supplement: Supplementary file 1 [file Table_1.docx]

**Supplemental Material: Glossary**

| **Term** | **Definition of the Term** | **Sources** |
| --- | --- | --- |
| Weakly Interacting Massive Particles (WIMPs) | are particles that are considered potential candidates for hot and cold dark matter in the universe. They are studied through measurements of their interactions with nuclei, including elastic and inelastic scatterings. | Ejiri, H. (2000). Nuclear spin isospin responses for low-energy neutrinos. *Physics Reports*, *338*(3), 265-351. <https://doi.org/10.1016/S0370-1573(00)00044-2> |
| Artificial intelligence (AI) | the ability of a digital computer or computer-controlled robot to perform tasks commonly associated with intelligent beings. | Encyclopaedia Britanica |
| Biomolecule: | A biomolecule is any of numerous substances produced by cells and living organisms. Biomolecules come in a wide range of sizes and structures and perform a vast array of functions. | Encyclopaedia Britanica |
| Bioactives: | Is a compound that has an effect on a living organism, tissue or cell. | Santé-Lhoutellier, V., & Ferraro, V. (2024). Contribution of bioactive compounds from meat. |
| Bioactive peptides (BAPs): | are made up of amino acids each ranging between 2 and 30 amino acids in length. | Mustafa, K., Kanwal, J., Musaddiq, S., Khakwani, S. (2020). Bioactive Peptides and Their Natural Sources. In: Egbuna, C., Dable Tupas, G. (eds) *Functional Foods and Nutraceuticals. Springer, Cham.* 75-97. https://doi.org/10.1007/978-3-030-42319-3_5 |
| Machine learning (ML): | is a process that empowers computers to learn independently by spotting patterns and making decisions based on data. It is especially useful when writing detailed instructions for every scenario is impractical. | Encyclopaedia Britanica |
| Deep learning (DL): | is a type of machine learning (ML) where neural networks have four or more layers, including the initial input and final output, that can discover features in data without initial prompting. | Encyclopaedia Britanica |
| Artificial Neural Network (ANN): | is a computer program designed to mimic the brain's neural network, enabling cognitive functions like problem-solving and machine learning. | Encyclopaedia Britanica |
| Molecular Docking (MD): | is a structure-based computational method that generates the binding mode and affinity between ligands and targets by predicting their interactions. | Sulimov, A.; Kutov, D.; Ilin, I.; Zheltkov, D.; Tyrtyshnikov, E.; Sulimov, V. Supercomputer docking with a large number of de grees of freedom. SAR QSAR Environ. Res., 2019, 30(10), 733-749. http://dx.doi.org/10.1080/1062936X.2019.1659412 PMID: 31547677 |
| Peptides Qualitative Structure-Activity Relationships (QSAR): | is a mathematical model relating one or more quantitative parameters, which are derived from the chemical structure to a quantitative measure of a property or activity. | ECHA (European Chemical Agency) (2016). Practical guide How to use and report (Q)SARs. Version 3.1. |
| Antioxidant peptides: | are naturally occurring or artificially designed peptides that can reduce the levels of ROS and other pro-oxidants, thus showing great potential in the treatment of oxidative stress-related diseases. | Zhu, Y., Wang, K., Jia, X., Fu, C., Yu, H., & Wang, Y. (2024). Antioxidant peptides, the guardian of life from oxidative stress. *Medicinal Research Reviews*, *44*(1), 275-364. <https://doi.org/10.1002/med.21986>. |
| Antioxidant | is a molecule (or an ion, or a relatively stable radical) that is capable of slowing or even preventing the oxidation of other molecules. | Pinchuk, I., Shoval, H., Dotan, Y., & Lichtenberg, D. (2012). Evaluation of antioxidants: scope, limitations and relevance of assays. *Chemistry and physics of lipids*, *165*(6), 638-647. |
| A qualitative structure–activity relationship (QSAR): | refers to the relationship between the structural features of molecules and their observed biological activities, where *qualitative QSAR* focuses on classifying compounds (e.g., active vs. inactive) based on structural patterns rather than predicting exact activity values. | Cherkasov, A. et al. (2014). *QSAR Modeling: Where have you been? Where are you going to?* *Journal of Medicinal Chemistry*, 57(12), 4977–5010. <https://doi.org/10.1021/jm4004285> |
| Internet of things (IoT) | refers to a network of physical devices, vehicles, appliances, and other physical objects that are embedded with sensors, software, and network connectivity, allowing them to collect and share data. | <https://www.ibm.com/think/topics/internet-of-things>. |
| Discrete wavelets transform (DWT): | is a transform that decomposes a given signal into a number of sets, where each set is a time series of coefficients describing the time evolution of the signal in the corresponding frequency band. | Nechyba, M. C. (2004). Introduction to the discrete wavelet transform (DWT). *University of Florida, February*. |
| Anti-inflammatory: | is the property of a substance (food, drugs etc.,) or treatment that reduces inflammation, fever or swelling | Harvard Health Publishing- Harvard Medical School |
| Functional foods: | are the foods which are enriched with any essential nutrients or to have an additional function by adding new ingredients or more of existing ingredients. They are fortified with essential minerals, vitamins etc, which are useful in reducing many health problems. | Academy of Nutrition and Dietetics (AND). [www.eatright.org](http://www.eatright.org). |
| Angiotensin-converting enzyme (ACE): | is a crucial enzyme in the renin-angiotensin system that regulates blood pressure by converting angiotensin I to angiotensin II, a potent vasoconstrictor. | National Library of Medicine- National Center for Biotechnology Information. [ACE angiotensin I converting enzyme [Homo sapiens (human)] - Gene - NCBI](https://www.ncbi.nlm.nih.gov/gene/1636). |
| Natural Language Processing (NLP) | Natural Language Processing (NLP) is a field that combines computer science, artificial intelligence and language studies. It helps computers understand, process and create human language in a way that makes sense and is useful. | [Natural Language Processing (NLP) - Overview - GeeksforGeeks](https://www.geeksforgeeks.org/nlp/natural-language-processing-overview/) |
| Branched-chain amino acids (BCAAs) | are a group of three essential amino acids that play key roles in muscle metabolism, energy production, and recovery. They are termed "branched chain" due to their chemical structure. | Wolfe, R. R. (2017). Branched-chain amino acids and muscle protein synthesis in humans: myth or reality? *Journal of the International Society of Sports Nutrition*, *14*(1), 30. |
| Immunomodulatory activities |  | Olayem et al. (2024). Immunomodulatory plant-based foods, it’s chemical, biochemical and pharmacological approaches. *Medicinal Plants-Chemical, Biochemical, and Pharmacological Approaches*. |
| Non-Communicable Diseases (NCDs) | also known as chronic diseases, tend to be of long duration and are the result of a combination of genetic, physiological, environmental and behavioural factors are of non-contagious nature. NCDs are cardiovascular diseases, cancers, chronic respiratory diseases and diabetes. | World Health Organization and National Library of Medicine |
| Convolutional Neural Networks (CNNs) | is a specialized type of deep learning algorithm mainly designed for tasks that necessitate object recognition, including image classification, detection, and segmentation. | An Introduction to Convolutional Neural Networks (CNNs). <https://www.datacamp.com/tutorial/introduction-to-convolutional-neural-networks-cnns>. |
| Recurrent Neural Networks (RNNs) | is a deep neural network trained on sequential or time series data to create a machine learning (ML) model that can make sequential predictions or conclusions based on sequential inputs | IBM |
| Glucagon-Like Peptide (GLP1) | Is a natural hormone made in the gut that plays an important role in managing appetite and blood sugar. GLP-1 peptides are small chains of amino acids that mimic the actions of GLP-1. | HOLST, JJ (2007). The Physiology of Glucagon-like Peptide 1. *Physiol*ogy *Rev*iew, 87: 1409–1439, 2007; doi:10.1152/physrev.00034.2006. |
| Free Fatty Acid Receptor (FFA1) | Free fatty acid receptor 1 (FFAR1, also known as GPR40) is a seven transmembrane G protein-coupled receptor (GPCR) highly expressed in pancreatic β-cells, intestinal L, K, and I cells, and in neurons. | [Bioorganic & Medicinal Chemistry Letters, 2021](https://www.sciencedirect.com/science/article/pii/S0960894X21001955). |
| Dipeptidy1 peptidase-IV (DPP-IV) | is a ubiquitous enzyme that acts on incretin hormones, mainly GLP-1 (glucagon-like peptide-1) and GIP (gastric inhibitory peptide), which maintain glucose homeostasis by increasing insulin secretion and decreasing glucagon secretion. | National Library of Medicine- National Center for Biotechnology Information |
| Methicillin-resistant Staphylococcus aureus (MRSA) | is a type of *Staphylococcus aureus* that is resistant to most beta-lactam antibiotics, antistaphylococcal penicillins (e.g., methicillin, oxacillin), and cephalosporins. | Babel BS, Decker CF. Microbiology and laboratory diagnosis of MRSA. Dis Mon. 2008 Dec;54(12):769-73. |
| Tumor T cell antigens (TTCA) | refer to antigens that are crucial for immunotherapy approaches, especially T cell-based therapies like checkpoint inhibitors, cancer vaccines, and adoptive T cell transfer and are recognized by T cells in the context of cancer. | Chen and Mellman (2017). *Elements of cancer immunity and the cancer- immune set point. Nature,* 18;541(7637):321-330. <https://doi.org/10.1038/nature21349>. |
| Quorum-Sensing (QS) | is a phenomenon in which microbial cells interact and communicate with each other by secreting some chemical molecules to which other cells respond and reciprocate to make a healthy conversation. | Nazir, R., Zaffar, M. R., & Amin, I. (2019). Bacterial biofilms: the remarkable heterogeneous biological communities and nitrogen fixing microorganisms in lakes. *Freshwater microbiology*, 307-340. |
| Gut Micro Biome (GM) | is the system of microorganisms that live in the digestive tract of animals, including humans. | MedicalNews Today (Gut microbiota: Definition, importance, and medical uses). https://www.medicalnewstoday.com/articles/307998. |
| Area Under the Receiver Operating Characteristic Curve (AUROC) | AUROC measures the ability of a binary classifier to distinguish between classes by calculating the area under the curve that plots the true positive rate against the false positive rate across all thresholds | Glassbox Medicine. *Measuring Performance: AUC (AUROC).* [https://glassboxmedicine.com/2019/02/23/measuring-performance-auc-auroc/](https://glassboxmedicine.com/2019/02/23/measuring-performance-auc-auroc/?utm_source=chatgpt.com) |
| Area Under the Precision–Recall Curve (AUPRC) | AUPRC quantifies the trade-off between precision and recall across classification thresholds and is particularly useful for evaluating classifiers on imbalanced datasets | Glassbox Medicine. *Measuring Performance: AUPRC.* [https://glassboxmedicine.com/2019/03/02/measuring-performance-auprc/](https://glassboxmedicine.com/2019/03/02/measuring-performance-auprc/?utm_source=chatgpt.com) |
| FAIR principles (Findable, Accessible, Interoperable, and Reusable) | The FAIR principles provide guidelines to ensure scientific data are findable, accessible, interoperable, and reusable for humans and machines | Wilkinson, M., Dumontier, M., Aalbersberg, I. *et al.* The FAIR Guiding Principles for scientific data management and stewardship. *Sci Data* **3**, 160018 (2016). https://doi.org/10.1038/sdata.2016.18 |
